# Burden of non-communicable diseases in Cyprus, 1990–2017: findings from the Global Burden of Disease 2017 study

**DOI:** 10.1186/s13690-021-00655-8

**Published:** 2021-07-29

**Authors:** Periklis Charalampous, Elena Pallari, Stefanos Tyrovolas, Nicos Middleton, Mary Economou, Brecht Devleesschauwer, Juanita A. Haagsma

**Affiliations:** 1 Department of Public Health, Erasmus MC University Medical Center, Rotterdam, The Netherlands; 2 Medical Research Council Clinical Trials Unit, Institute of Clinical Trials and Methodology, University College London, London, UK; 3 Health Services Research Center, Strovolos, Nicosia, Cyprus; 4 Parc Sanitari Sant Joan de Déu, Universitat de Barcelona, Barcelona, Spain; 5 Instituto de Salud Carlos III, Centro de Investigación Biomédica en Red de Salud Mental, CIBERSAM, Madrid, Spain; 6 School of Nursing, Hong Kong Polytechnic University, Hong Kong, SAR China; 7 Department of Nursing, School of Health Sciences, Cyprus University of Technology, Limassol, Cyprus; 8 Department of Epidemiology and Public Health, Sciensano, Brussels, Belgium; 9 Department of Veterinary Public Health and Food Safety, Ghent University, Merelbeke, Belgium

**Keywords:** Cyprus, Burden of disease, Non-communicable diseases

## Abstract

**Background:**

Non-communicable diseases (NCDs) accounted for over 90% of all deaths in the Cypriot population, in 2018. However, a detailed and comprehensive overview of the impact of NCDs on population health of Cyprus over the period of 1990 to 2017, expressed in disability-adjusted life years (DALYs), is currently not available. Knowledge about the drivers of changes in NCD DALYs over time is paramount to identify priorities for the prevention of NCDs in Cyprus and guide evidence-based decision making. The objectives of this paper were to: 1) assess the burden of NCDs in terms of years of life lost (YLLs), years lived with disability (YLDs), and DALYs in Cyprus in 2017, and 2) identify changes in the burden of NCDs in Cyprus over the 28-year period and assess the main drivers of these changes.

**Methods:**

We performed a secondary database descriptive study using the Global Burden of Disease (GBD) 2017 results on NCDs for Cyprus from 1990 to 2017. We calculated the percentage change of age-standardized DALY rates between 1990 and 2017 and decomposed these time trends to assess the causes of death and disability that were the main drivers of change.

**Results:**

In Cyprus in 2017, 83% (15,129 DALYs per 100,000; 12,809 to 17,707 95%UI) of total DALYs were due to NCDs. The major contributors to NCD DALYs were cardiovascular diseases (16.5%), neoplasms (16.3%), and musculoskeletal disorders (15.6%). Between 1990 and 2017, age-standardized NCD DALY rates decreased by 23%. For both males and females, the largest decreases in DALY rates were observed in ischemic heart disease and stroke. For Cypriot males, the largest increases in DALY rates were observed for pancreatic cancer, drug use disorders, and acne vulgaris, whereas for Cypriot females these were for acne vulgaris, psoriasis and eating disorders.

**Conclusion:**

Despite a decrease in the burden of NCDs over the period from 1990 to 2017, NCDs are still a major public health challenge. Implementation of interventions and early detection screening programmes of modifiable NCD risk factors are needed to reduce occurrence and exacerbation of leading causes of NCDs in the Cypriot population.

**Supplementary Information:**

The online version contains supplementary material available at 10.1186/s13690-021-00655-8.

## Background

Non-communicable diseases (NCDs) are a major cause of death and disability worldwide [1]. The four major groups of NCDs, namely cardiovascular diseases (CVDs), neoplasms, chronic respiratory diseases, and diabetes, cause over 70% of global mortality each year [1]. In Cyprus, a high-income country and a member state of the European Union (EU), NCDs have posed an even greater burden on population health. A 2018 World Health Organization (WHO) report revealed that NCDs accounted for over 90% of all deaths in the Cypriot population [1]. Markedly, neoplasms accounted for 19% of all deaths in Cyprus [2]. Moreover, the prevalence of diabetes in the Cypriot population was estimated to be 10% which is slightly higher compared to the prevalence of diabetes in other EU countries [3]. Additionally, Cyprus has the highest relative disease burden from diabetes (4.6%), although publishes proportionally less (1.2%) with almost half of research output dedicated to Type 2 diabetes, compared to the rest of the European countries (including the UK) [2]. Furthermore, smoking, physical inactivity and childhood obesity are some of the main public health risks identified in previous studies [2, 4, 5] as contributing to the growing NCD epidemic. Cyprus has a relatively centralized and co-ordinated vaccination system at the national level, with programmes targeting a covering at nearly 100% [6]. Therefore, the contributing burden of disease on mortality and morbidity are heavily based on NCDs. WHO member states have endorsed a set of policy options and cost-effective NCD interventions that can be used to tackle the burden of NCDs [7]. Furthermore, population ageing is causing an increase in the burden of specific health conditions; WHO’s Global Strategy and Action Plan on Ageing and Health 2016–2020 [8] urges countries to establish intervention policies on healthy population ageing. Understanding at which ages the NCD burden starts to accumulate may shed light on how to introduce better policies and hence, how to reduce the projected NCD burden. The economic growth and prosperity in Cyprus (ranked 33th out of 167 countries) with health and living conditions being at the top of the indicators list, may be responsible for the reduction of poverty [6], but may have contributed to the increase of obesity as a side-effect of such a wealthy lifestyle. To exacerbate, as Cyprus has life expectancy at 82.2 years of age (amongst the highest life expectancy at birth between European countries) [9], the ageing population is more likely to have more than one chronic disease, with further imposed health burdens for the individual and healthcare associated costs to the society.

However, resources are limited and it is therefore important for policy makers to have up to date quantifications of the impact of NCDs on the population health of Cyprus and the relative attributes of modifiable risk factors to guide priority setting.

The impact of NCDs on population health can be quantified using mortality or incidence and prevalence. However, NCDs are characterized by heterogeneity in health outcomes, with great variety in severity, duration and mortality rates [10]. Moreover, with increasing life span, information on disability has become more important. Summary measures of population health (SMPH) combine mortality, morbidity and disability into one single index [11, 12]. This allows for comparison between distinct health outcomes, and subsequently, comparison of the population health impact of a range of diseases and risk factors. SMPHs are therefore vital tools for priority setting purposes [11, 12]. A widely used SMPH is the Disability-Adjusted Life Year (DALY) [10]. The DALY-concept integrates premature mortality in years of life lost (YLLs) and morbidity in years lived with disability (YLDs) [11, 12]. The DALY has been used in the landmark Global Burden of Disease (GBD) studies which aim to assess up to date country-specific incidence, prevalence, mortality, YLLs, YLDs, and DALYs for over 300 diseases and injuries in 195 countries and territories using a systematic analysis [13–15]. The approach that is used by the GBD researchers ensures that the calculated incidence, prevalence, mortality, YLLs, YLDs, and DALYs are comparable and internally consistent across years and regions.

Annually, updated methods and results of the GBD study are published. However, a detailed and comprehensive overview of the burden of disease of NCDs in Cyprus is currently not available. Investigation of time trends and decomposition of these time trends can pinpoint the diseases that contributed most to the change in burden of disease of NCD. Knowledge about the main drivers of changes in NCD YLDs, YLLs, and DALYs over time is imperative for health professionals and policy makers to identify priorities for the prevention of NCDs in Cyprus and guide evidence-based decision making. Until recently, the health authorities in Cyprus lacked knowledge about the population’s state of health and health policies have not been targeted. In fact, it was shown that the biomedical research funded on the island does not correspond to the DALYs of the Cypriot population [2]. Therefore, assessing the burden of NCDs in Cyprus is an important topic for public health policy planning for primary health interventions and prioritizing NCD prevention policies.

Here, we have sought to provide a comprehensive overview of the age-standardized YLLs, YLDs and DALYs of NCDs in Cyprus in 2017, to investigate rates of change over the period from 1990 to 2017, and to identify the NCDs that were main drivers of these changes. Comparison of NCD-related DALYs between age-specific and age-standardized rates was also made.

## Methods

### Overview

We performed a secondary database descriptive study using the GBD 2017 results. The GBD 2017 study analyzed the impact of 359 diseases and injuries across 23 age groups and both sexes, and 195 countries and territories between 1990 and 2017 [15]. Detailed descriptions on the GBD study methodology, data, and analysis have been previously described [15, 16]. In this study we restricted our analysis to YLLs, YLDs, and DALYs due to NCDs in the Cypriot population from 1990 to 2017. Briefly, YLLs are calculated by multiplying the number of deaths by the global standard life expectancy at that age. YLDs are calculated as prevalence of a health state multiplied by the corresponding disability weight of this health state. YLD estimates are corrected for comorbidity using methods described elsewhere in the GBD study [15]. DALYs are calculated by adding the YLLs and YLDs, thereby incorporating both mortality and morbidity [13–16]..

The diseases studied by GBD are arranged in standard hierarchical categories of four levels. Level 1 causes consist of three categories, namely: communicable, maternal, neonatal, and nutritional diseases (Group I); NCDs (Group II); and injuries (Group III). Each Level can be broken down into a more detailed classification. For example, Group II can be broken down into 12 different diseases at Level 2. The NCD categories (at Level 2 of the cause hierarchy) featured in the GBD 2017 study were neoplasms, cardiovascular diseases, chronic respiratory diseases, digestive diseases, neurological disorders, mental disorders, substance use disorders (SUDs), diabetes and chronic kidney disease (CKD), skin and subcutaneous diseases, sense organ diseases, musculoskeletal disorders, and other NCDs. If the interest is for DALYs on CVDs, for example, then these causes can be further broken down into 11 sub-diseases (Level 3) and more details (Level 4). The GBD 2017 disease categories by level can be found elsewhere [17]. For the present analysis, we report Level 2 and Level 3 NCD (sub-)causes (see Additional file 1).

Cyprus has been divided into two parts; the northern part which is under Turkish occupation and the southern or government-controlled part which consists of five districts, namely Nicosia, Ammochostos, Larnaca, Limassol, and Paphos. The total population in the government-controlled area is estimated at 875,900 in 2018 [18]. All districts of Cyprus will be referred to as a whole in the rest of the manuscript.

### Source of data and presentation

In our study, we analyzed and reported levels and trends of age-standardized YLL, YLD, and DALY rates. An age-standardized rate is a weighted average of the age-specific rates per 100,000 of population, where the weights are the proportions of the standard population in the corresponding age groups. Cause-specific mortality was informed primarily from vital registration data. Epidemiological data from scientific reports and health surveys were used to generate NCD-specific prevalence and incidence estimates. YLL and YLD estimates due to NCDs were adjusted for incompleteness and misclassification using standardized approaches [15, 16]. YLLs, YLDs, and DALYs were provided by the visualisation “*GBD Results*” tool (Institute for Health Metrics and Evaluation (IHME), 2017; available online at: http://ghdx.healthdata.org/gbd-results-tool).

Age-standardized DALY rates, and its components YLL and YLD, related to all NCDs in Cyprus from 1990 to 2017, were analyzed for both genders at Levels 2 and 3. We, first, calculated the proportion of the top five Level 2 and Level 3 NCDs that contributed most to the burden of NCD DALYs, YLLs, and YLDs in Cyprus in 2017 using the cross-multiplication method, known as ‘*rule of three’* [19]. For changes over time, we presented the percentage of change for each age-standardized YLL, YLD, and DALY rate in 1990 and 2017. A positive percentage change indicates an increase, whereas a negative change a decrease from 1990 to 2017. Additionally, we decomposed differences in the DALY-related NCDs over the period from 1990 to 2017 to assess the main drivers of change in DALY, YLL and YLD rates of NCDs. Finally, we have examined the age distribution of NCD DALYs and we reported the NCD DALY rates for the elderly (70+ years) category over the 28-year study period.

### Uncertainty

Uncertainty distribution for each NCD outcome variable (YLL, YLD, and DALY) was captured and propagated by 1000 draws from the posterior distributions. The results for each variable of interest were derived from the mean of 1000 draws and the 95% uncertainty intervals (UIs) were derived from the 2.5th and 97.5th percentiles of the corresponding draws of the sampled YLL, YLD, and DALY variables [20, 21].

## Results

### Burden of disease of NCDs, in Cyprus in 2017

In Cyprus in 2017, the total burden of disease was 18,287 DALYs (15,607 to 21,322 95%UI) per 100,000 of which 83% (15,129 DALYs per 100,000; 12,809 to 17,707 95%UI) were due to NCDs.

The top three Level 2 causes of NCDs in Cyprus in terms of DALYs were CVDs, neoplasms, and musculoskeletal disorders. CVDs accounted for 16.5% (2497 DALYs per 100,000; 2276 to 2729 95%UI), neoplasms for 16.3% (2469 DALYs per 100,000; 2277 to 2681 95%UI), and musculoskeletal conditions for 15.6% (2365 DALYs per 100,000; 1719 to 3177 95%UI) of NCD DALYs. Mental disorders and neurological disorders were also major causes of the NCD DALYs in 2017 in Cyprus; mental disorders were responsible for 11.4% of NCD DALYs (1732 DALYs per 100,000; 1284 to 2239 95%UI), and neurological disorders for 9.9% (1503 DALYs per 100,000; 1136 to 1932 95%UI). Together, the afore-mentioned NCD health conditions contributed approximately two-thirds of the NCD DALYs in Cyprus. The leading Level 3 NCD causes due to DALYs were low back pain (9.3%; 1405 DALYs per 100,000; 1001 to 1902 95%UI) and ischemic heart disease (IHD; 9.0%; 1376 DALYs per 100,000; 1241 to 1538 95%UI). Headache disorders were responsible for 6.0% (909 DALYs per 100,000; 600.4 to 1294 95%UI) and 5% of total NCD DALYs were due to diabetes (756 DALYs per 100,000; 601.8 to 943.7 95%UI).

**Fig. 1:**
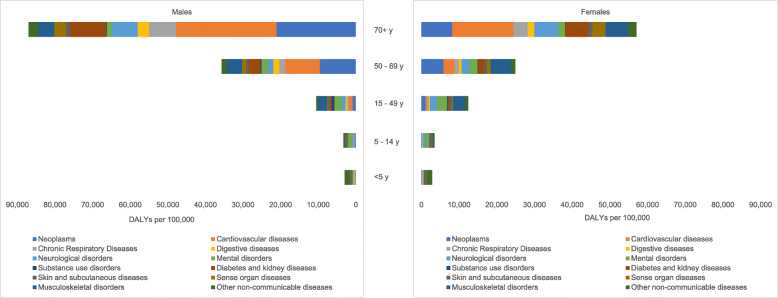
DALYs per 100,000 due to NCD Level 2 causes by age-category and sex in Cyprus, 2017

Figure 1 presents the number of DALYs per 100,000 due to NCDs in Cyprus in 2017 by age-category and sex.

Overall, the NCD DALY rates per 100,000 were similar in males (16,627 DALYs per 100,000; 14,357 to 19,159 95%UI) and females (13,923 DALYs per 100,000; 11,453 to 16,802 95%UI). However, in the older age categories from age 50 and older the higher NCD DALY rates among males compared to females stand out. In addition, Fig. 1 shows that NCD DALY rates increase by age-category, with highest NCD DALY rates per 100,000 among the 70+ years old (males: 87,037 DALYs (78,212–96,049 95%UI); females: 57,096 DALYs (50,877–68,980 95%UI). The leading causes of NCD DALYs in this age-category were CVDs and neoplasms.

In 2017, YLLs were responsible for 42% of the NCD DALYs (6345 YLLs per 100,000; 5892 to 6856 95%UI). The leading Level 2 causes of NCD YLLs were neoplasms (36.7%; 2327 YLLs per 100,000; 2150 to 2523 95%UI) and CVDs (34.2%; 2171 YLLs per 100,000; 1981 to 2389 95%UI). 7.2% of the NCD YLLs came from diabetes and CKD (460.2 YLLs per 100,000; 417.4 to 505.6 95%UI), and 6.2% from neurological disorders (391.8 YLLs per 100,000; 362.2 to 423.6 95%UI). The leading Level 3 causes of NCD YLLs were IHD (20.7%; 1314 YLLs per 100,000; 1187 to 1475 95%UI), lung cancer (7.3%, 461.2 YLLs per 100,000; 412.4 to 515.5 95%UI) and stroke (6.4%; 406.6 YLLs per 100,000; 368.1 to 456.6 95%UI).

YLDs were responsible for 58% of the overall NCD DALYs (8785 YLDs per 100,000; 6592 to 11,303 95%UI). The leading Level 2 causes of NCD YLDs were musculoskeletal disorders (26.6%; 2337 YLDs per 100,000; 1692 to 3152 95%UI), mental disorders (19.7%; 1732 YLDs per 100,000; 1283 to 2239 95%UI) and neurological disorders (12.6%; 1111 YLDs per 100,000; 748 to 1543 95%UI). Meanwhile, the leading Level 3 causes of NCD YLDs were low back pain (1405 YLDs per 100,000; 1001 to 1902 95%UI) and headache disorders (909 YLDs per 100,000; 600.4 to 1294 95%UI), contributing 16.0 and 10.3% to the total NCD YLDs, respectively. Other NCD-groups that contributed a notable amount to NCD YLDs were depressive disorders (5.5%; 482.2 YLDs per 100,000; 342.6 to 658.9 95%UI) and anxiety disorders (5.4%; 472.3 YLDs per 100,000; 335.9 to 628.4 95%UI). Table 1 shows the rankings for the top five Level 2 and Level 3 NCDs that contributed most to overall NCD YLLs, YLDs, and DALYs in Cyprus in 2017.

**Table 1:** The contribution of the top five Level 2 and Level 3 causes to overall NCD YLLs, YLDs and DALYs in Cyprus in 2017

**Rank**	**Level 2** **NCD**	**DALY NCD** **(%)**	**Level 2** **NCD**	**YLL NCD** **(%)**	**Level 2** **NCD**	**YLD NCD** **(%)**
**1**	CVDs	16.5%	Neoplasms	36.7%	Musculoskeletal disorders	26.6%
**2**	Neoplasms	16.3%	CVDs	34.2%	Mental disorders	19.7%
**3**	Musculoskeletal disorders	15.6%	Diabetes and CKD	7.6%	Neurological disorders	12.6%
**4**	Mental disorders	11.4%	Neurological disorders	6.2%	Skin diseases	7.2%
**5**	Neurological disorders	9.9%	Chronic respiratory diseases	4.9%	Other NCDs	6.8%
**Rank**	**Level 3** **NCD**	**DALY NCD** **(%)**	**Level 3** **NCD**	**YLL NCD** **(%)**	**Level 3** **NCD**	**YLD NCD** **(%)**
**1**	Low back pain	9.3%	IHD	20.7%	Low-back pain	16.0%
**2**	IHD	9.0%	Lung cancer	7.3%	Headache disorders	10.3%
**3**	Headache disorders	6.0%	Stroke	6.4%	Depressive disorders	5.5.%
**4**	Diabetes	5.0%	Diabetes	4.5%	Neck pain	5.5%
**5**	Stroke	3.5%	Alzheimer’s disease	3.9%	Anxiety disorders	5.4%

*CKD* chronic kidney disease, *CVDs* cardiovascular diseases, *DALY* disability-adjusted life years, *IHD* ischemic heart disease, *NCD* non-communicable disease, *YLL* years of life lost, *YLD* years lived with disability

### Changes in NCD DALY rates in Cyprus, 1990–2017

Table 2 shows the age-standardized YLL, YLD, DALY rates and percentage change for Level 2 NCD-group, in Cyprus between 1990 and 2017.

**Table 2:** Age-standardized YLL, YLD, DALY rates and percentage change for Level 2 NCD-group, in Cyprus from 1990 to 2017

	YLLs			YLDs			DALYs		
	1990 age-standardized rates per 100,000	2017 age-standardized rates per 100,000	Percentage change in age-standardized rates per 100,000	1990 age-standardized rates per 100,000	2017 age-standardized rates per 100,000	Percentage change in age-standardized rates per 100,000	1990 age-standardized rates per 100,000	2017 age-standardized rates per 100,000	Percentage change in age-standardized rates per 100,000
**All causes**	**14,614 (14,339 - 14,887)**	**7904 (7332 - 8540)**	**−45.9%**	**10,597 (7972 - 13,641)**	**10,383 (7841 - 13,374)**	**− 2.0%**	**25,211 (22,605 - 28,229)**	**18,287 (15,607 - 21,322)**	**−27.5%**
**Group II: Non-communicable diseases**	**10,793 (10,484 - 11,096)**	**6345 (5892 - 6856)**	**−41.2%**	**8815 (6608 - 11,395)**	**8785 (6592 - 11,303)**	**− 0.3%**	**19,608 (17,362 - 22,248)**	**15,129 (12,809 - 17,707)**	**−22.8%**
**Cardiovascular diseases**	4583 (4428 - 4752)	2171 (1981- 2389)	−52.6%	397.1 (291.5–516.6)	326.2 (241–420.6)	−17.9%	4980 (4787 - 5183)	2497 (2276- 2729)	−49.9%
**Neoplasms**	2718 (2592 - 2828)	2327 (2150- 2523)	−14.4%	81.7 (59.8–105.7)	142.2 (103.3–189.4)	74.1%	2800 (2673 - 2916)	2469 (2277- 2681)	−11.8%
**Musculoskeletal disorders**	41.9 (30.3–51.3)	27 (20–32)	−34.7%	2337 (1700 - 3151)	2337 (1692 - 3152)	0.0%	2379 (1740 - 3187)	2365 (1719 - 3177)	−0.6%
**Mental disorders**	0.02 (0.01–0.02)	0.04 (0.02–0.05)	100.0%	1734 (1290 - 2246)	1732 (1283- 2239)	−0.1%	1734 (1290 - 2246)	1732 (1284 - 2239)	−0.1%
**Neurological disorders**	543.2 (500.6–578.8)	391.8 (362.2–423.6)	−27.9%	1117 (761–1526)	1111 (748–1543)	−0.5%	1660 (1310 - 2072)	1503 (1136 - 1932)	−9.4%
**Diabetes and kidney diseases**	825.7 (758.4–901.3)	460.2 (417.4–505.6)	−44.3%	494.9 (348.6–680)	539.8 (373–734.9)	9.1%	1321 (1156 - 1512)	1000 (832–1206)	− 24.3%
**Other non-communicable diseases**	1020 (725–1272)	298.5 (262.1–333.6)	−70.7%	679.6 (466.5–959.7)	595.6 (408.8–836.7)	−12.4%	1699 (1314 - 2095)	894 (706.4–1133)	−47.4%
**Chronic respiratory diseases**	519.2 (460.3–576.9)	311.2 (276.1–344.2)	−40.0%	484.5 (374.5–605.4)	449.1 (352.8–556.9)	−7.3%	1004 (880–1138)	760.7 (659.9–870.2)	− 24.2%
**Skin and subcutaneous diseases**	19.7 (8.9–27.5)	17.1 (8.8–21.7)	−13.2%	564.9 (383.1–809.4)	632.3 (433.7–902.8)	11.9%	584.7 (403.1–825.9)	649.4 (449.4–916.9)	11.1%
**Sense organ diseases**	0	0	N/A	531.5 (359.3–752.3)	501.8 (339.8–715.5)	− 5.6%	531.5 (359.3–752.3)	501.8 (339.8–715.5)	−5.6%
**Digestive diseases**	474.1 (380.6–570.6)	270.6 (241.7–302.6)	−42.9%	204.5 (142.3–279.5)	217.4 (152–298)	6.3%	678.7 (557.4–799.7)	488 (419.4–576.7)	−28.1%
**Substance use disorders**	48 (37.9–61.2)	69.3 (59–84)	44.1%	188.1 (131.1–252.3)	199.7 (138.3–266.2)	6.2%	236.2 (178–298.7)	268.9 (208.8–333.9)	13.9%

Data in parentheses are 95% uncertainty intervals (UIs)

A positive percentage change indicates an increase and a negative change a decrease between 1990 and 2017

*DALYs* disability-adjusted life years, *YLLs* years of life lost, *YLDs* years lived with disability, *N/A* not applicable

**Fig. 2:**
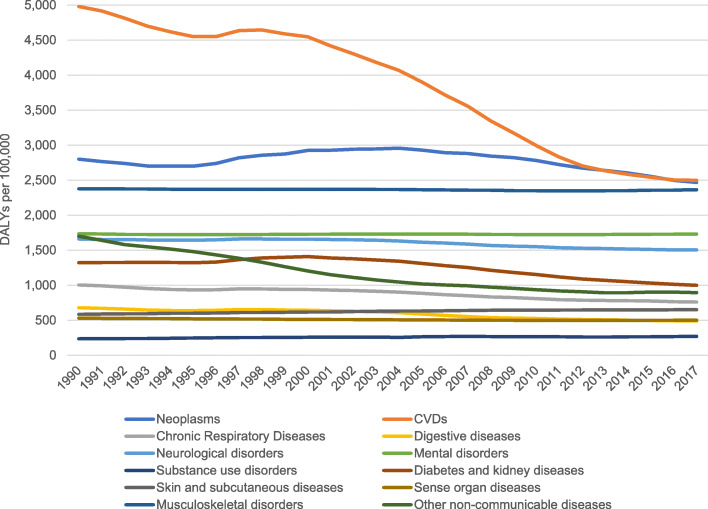
Age-standardized DALY rates of Level 2 NCDs per 100,000 in Cyprus from 1990 to 2017

Over the period from 1990 to 2017 NCD DALY rates in Cyprus decreased from 19,608 DALYs per 100,000 (17,362 to 22,248 95%UI) in 1990 to 15,129 DALYs per 100,000 (12,809 to 17,707 95%UI) in 2017, representing a 23% decrease over the 28-year study period. Time trends by cause of NCD varied considerably. Overall, CVDs and neoplasms had the highest DALY rates over the 28-year study period. However, CVD DALYs decreased between 1990 and 1995, followed by a slight growth until 1998, while from 1999 to 2017 they decreased steeply. DALY rates due to neoplasms increased between the 1995–2005 period, followed by a decreasing trend until 2017. A similar pattern was also seen in diabetes and CKD. On the other hand, more gradual decreases in DALY rates were observed for neoplasms, neurological disorders, diabetes and kidney diseases, other non-communicable disease and chronic respiratory diseases. Figure. 2 represents how NCD DALY rates has changed over the period from 1990 to 2017 in Cyprus; the figure is given as age-standardized DALYs per 100,000.

Age-standardized NCD DALY rates for males decreased significantly, from 21,012 DALYs (18,937 to 23,505 95%UI) in 1990 to 16,627 DALYs per 100,000 (14,358 to 19,159 95%UI) in 2017, a decrease of 20.9% (− 4385 DALYs per 100,000). Largest absolute decreases in NCD DALY rates were observed for CVDs (− 2814 DALYs per 100,000), of which IHD (− 1944 DALYs per 100,000) and stroke (− 615 DALYs per 100,000) ranked among the most significant causes of decreases in DALY rates. Congenital birth defects (− 580 DALYs per 100,000), chronic obstructive pulmonary disease (COPD; − 192 DALYs per 100,00) and diabetes (− 124 DALYs of 100,000) also showed large decreases in DALY rates over the period from 1990 to 2017. On the other hand, DALYs associated with pancreatic cancer (+ 55 DALYs per 100,00), drug use disorders (+ 53 DALYs per 100,000), acne vulgaris (+ 31 DALYs per 100,000), and kidney cancer (+ 26 DALYs per 100,000) increased from 1990 to 2017 (see Additional file 2).

For Cypriot females age-standardized NCD DALY rates declined from 18,527 DALYs (16,049 to 21,367 95%UI) in 1990 to 13,923 DALYs per 100,000 (11,453 to 16,802 95%UI) in 2017, reflecting a decrease of 24.8% (− 4604 DALYs per 100,000). The leading causes of the absolute decrease in NCD DALY rates in females were IHD (− 1150 DALYs per 100,000), stroke (− 657 DALYs per 100,000), and diabetes mellitus (− 342 DALYs per 100,000). An increase in age-standardized DALY rates were observed for acne vulgaris (+ 42 DALYs per 100,000), psoriasis (+ 28 DALYs per 100,000), and eating disorders (+ 25 DALYs per 100,000) over the period of 1990–2017 (see Additional file 2).

### Changes in NCD YLL rates in Cyprus, 1990–2017

In 1990, YLLs due to NCDs constituted 55% (10,793 YLLs per 100,000; 10,484 to 11,096 95%UI) of the overall NCD DALYs (19,608 DALYs per 100,000; 17,362 to 22,248 95%UI), whereas in 2017 they accounted for 42% (6345 YLLs per 100,000; 10,793 to 11,096 95%UI) of the overall NCD burden (15,129 DALYs per 100,000; 12,809 to 17,707 95%UI) (Table 2).

For males age-standardized NCD YLL rates declined from 13,088 YLLs (12,629 to 15,539 95%UI) in 1990 to 8610 YLLs per 100,000 (7747 to 9546 95%UI) in 2017 (− 34.2%; − 4478 NCD YLLs per 100,000). Key contributors that led to the NCD YLLs reductions were IHD (− 1913 YLLs), stroke (− 606 YLLs per 100,000), congenital birth defects (− 578 YLLs per 100,000), and diabetes (− 217 YLLs per 100,000). On the other hand, YLL rates associated with pancreatic cancer, drug use disorders, kidney cancer, and liver cancer increased by 55, 40, 25, and 19 YLLs per 100,000 between 1990 and 2017 (see Additional file 2).

For females NCD YLL rates decreased by 50.4%, from 8812 YLLs (8520 to 9104 95%UI) in 1990 to 4372 YLLs per 100,000 (3961 to 4859 95%UI) in 2017. The main contributors to this decrease were IHD (− 1125 YLLs per 100,000), stroke (− 646 YLLs per 100,000), congenital birth defects (− 472 YLLs per 100,000), and diabetes mellitus (− 361 YLLs per 100,000). Over the same period, the DALY rates of lung cancer (+ 19 YLLs per 100,000) and pancreatic cancer (+ 18 YLLs per 100,000) increased (see Additional file 2).

### Changes in NCD YLD rates in Cyprus, 1990–2017

Between 1990 and 2017, the share of NCD YLDs of the total NCD burden increased by 13%. Specifically, in 1990, NCD YLDs were responsible for 45% (8815 YLDs per 100,000; 6608 to 11,395 95%UI) of the overall NCD DALYs (19,608 DALYs per 100,000; 17,362 to 22,248 95%UI), while in 2017 NCD YLDs were responsible for 58% (8785 YLDs per 100,000; 6592 to 11,303 95%UI) of the overall NCD burden (15,129 DALYs per 100,000; 12,809 to 17,707 95%UI), (Table 2).

Between 1990 and 2017 the NCD YLD rates for Cypriot males increased by 92 YLDs per 100,000. Major contributors to this increase were observed for diabetes (+ 93 YLDs per 100,000) and neoplasms (+ 79 YLDs per 100,000). Notably, within the neoplasms category, the YLD rates of prostate cancer (+ 34 YLDs per 100,000) and colorectal cancer (+ 11 YLDs per 100,000) showed the largest increase. Age-standardized YLD rates of oral disorders (− 35 YLDs per 100,000), IHD (− 31 YLDs per 100,000), and asthma (− 23 YLDs per 100,000) showed the largest decreases in YLD rates (see Additional file 2).

For females, between 1990 and 2017, the NCD YLD rates decreased by 166 YLDs per 100,000. The largest decreases were observed for oral disorders (− 43 YLDs per 100,000), hemoglobinopathies and haemolytic anemias (− 33 YLDs per 100,000), gynecological diseases (− 28 YLDs per 100,000), and COPD (− 25 YLDs per 100,000). The largest increases were seen in the YLD rates of neoplasms (+ 44 YLDs per 100,000), acne vulgaris (+ 42 YLDs per 100,000), psoriasis (+ 27 YLDs per 100,000), eating disorders (+ 25 YLDs per 100,000) and diabetes mellitus (+ 19 YLDs per 100,000), between 1990 and 2017 (see Additional file 2).

### Changes in NCD DALY rates in the elderly (70+ years) in Cyprus, 1990–2017

Over the period from 1990 to 2017, the NCD DALY rates in the elderly (70+ years) were higher compared to the age-standardized NCD-related DALYs per 100,000 of other age groups. Major contributors to the NCD DALYs in elderly in 2017 were CVDs, neoplasms, and diabetes and CKD. CVDs accounted for 27.5% (20,890 DALYs per 100,000; 19,253 to 22,783 95%UI), neoplasms for 18% (13,950 DALYs per 100,000; 12,687 to 15,184 95%UI), and diabetes and CKD for 10% (7761 DALYs per 100,000; 6808 to 8847 95%UI) of the total NCD burden. Leading Level 3 causes of NCDs in the elderly in terms of DALYs were IHD (13.5%; 10,296 DALYs per 100,000; 9384 to 11,649 95%UI) and diabetes (7%; 5506 DALYs per 100,000; 4663 to 6434 95%UI). Stroke was also a major cause in the elderly contributing 7% (5299 DALYs per 100,000; 4749 to 5959 95%UI) to the overall NCD burden.

**Fig. 3:**
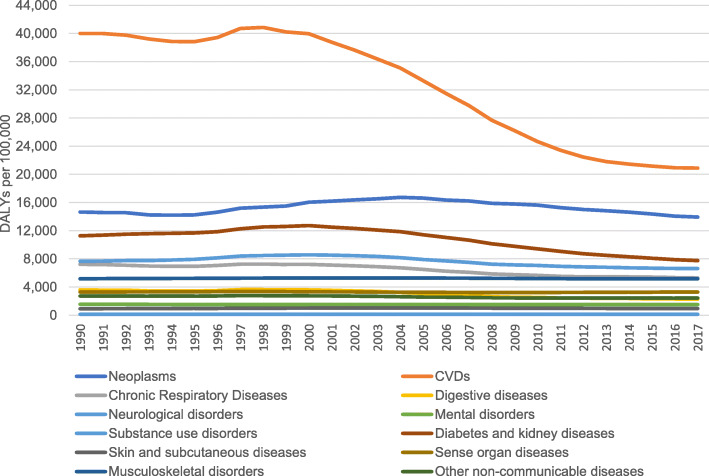
NCD DALY rates in elderly (70+ years) in Cyprus from 1990 to 2017

Between 1990 and 2017, NCD DALY rates in the elderly decreased from 98,331 DALYs per 100,000 (92,636 to 104,594 95%UI) in 1990 to 70,432 DALYs per 100,000 (63,850 to 77,228 95%UI) in 2017, representing a 28% decline. Time trends in NCD DALYs in the elderly over the 1990–2017 period varied considerably. CVDs and neoplasms DALYs showed the largest decline over the 1990–2017 period. However, CVD DALYs decreased between 1990 and 1995, followed by a slight increase between 1996 and 1998, and a rapid decline from 1999 to 2017. Neoplasm-related DALYs in the elderly slightly increased between 1995 and 2004, followed by a decrease between 2005 and 2017. The CVD DALY rates decreased sharply (− 47.8%) from 40,014 DALYs per 100,000 (38,152 to 42,251 95%UI) in 1990 to 20,890 DALYs per 100,000 (19,253 to 22,783 95%UI) in 2017. A similar pattern was also seen in DALYs due to diabetes; from 11,285 DALYs per 100,000 (9976 to 12,614 95%UI) in 1990 to 7761 DALYs per 100,000 (6808 to 8847 95%UI) in 2017, representing a decrease of 31%. More gradual decreases in NCD DALY rates among the elderly population were observed for neoplasms, digestive diseases, mental and neurological disorders, chronic respiratory diseases, and other non-communicable diseases. Figure 3 shows the NCD DALY rates in the elderly population over the period from 1990 to 2017.

## Discussion

### Summary of findings

The findings of this study showed that in Cyprus in 2017 83% of the total burden of disease was attributable to NCDs, and that CVDs, neoplasms and musculoskeletal disorders were the top contributors to the burden of NCDs. Between 1990 and 2017, age-standardized NCD DALY rates decreased by 23%. For both males and females, the largest decreases in DALY rates were observed in IHD and stroke. However, over this 28-year period, CVDs, neoplasms, and musculoskeletal disorders were consistently major contributors to NCD DALYs for both males and females. In particular, neoplasms and CVDs were mostly driven by YLLs, whereas musculoskeletal disorders were driven by YLDs. From 1990 to 2017, NCD DALY rates were highest among the elderly (70+ years).

Although the NCD burden of IHD and stroke, as quantified by age-standardized DALY rates, declined for both Cypriot males and females over the period from 1990 to 2017, they remained the main CVD DALY contributors. A possible explanation for this may be that around 30% of Cypriots have untreated hypertension [22]. Since hypertension is a major risk factor for IHD and stroke, the high prevalence of hypertension may therefore be a main contributor to the burden of IHD and stroke. Also, smoking is a well-established risk factor for IHD and stroke. Notably, the prevalence of tobacco use over time is consistently high in Cyprus compared with other European countries [23] and high IHD and stroke DALY rates may reflect the legacy of high cigarette smoking rates in Cyprus. To achieve further decreases in the prevalence and mortality of IHD and stroke in Cyprus it is therefore important to target these risk factors. For instance, by setting up early detection screening programmes for hypertension, and scaling up of smoking cessation interventions to prevent the disease burden possibly attributable to the tobacco use among the Cypriot population.

The proportion of DALY for diabetes in Cyprus was higher than in the WHO European Region [2]. Our findings showed that YLDs caused by diabetes increased for Cypriot males as well as females, during the period of 1990–2017. Environmental, lifestyle and genetic factors might have a significant effect on the pathogenesis of diabetes in the Cypriot population [24, 25]. Thus, the large proportion of diabetes-related DALY in the total NCD burden, as found in this study, calls for efforts to investigate leading risk factors for diabetes and kidney disease in Cyprus as well as improvements in diabetes management.

Cypriot males had substantially more YLLs and DALYs due to pancreatic cancer than females. Alcohol consumption and tobacco use have been identified as major risk factors for pancreatic cancer [26, 27]. In Cyprus, the smoking prevalence is high; similarly, the prevalence of heavy episodic drinking is estimated to be 28%, which is close to that of WHO EU countries (30%) [28]. However, a high proportion of alcohol-related and smoking-related disease burden increase the risk of other health conditions, such as CVDs and neoplasms. Smoking cessation and alcohol abuse interventions have been shown to be effective with a potential effect on public health [29, 30]. Thus, alcohol and tobacco control policies should also be considered in the Cypriot primary health care. Also, during the period from 1990 to 2017, the burden of SUDs increased for Cypriot males. This burden is mainly driven by alcohol and drug use disorders. Previous studies have yielded evidence of an association between alcohol consumption and/or drug use and unemployment status among males [31, 32]. Between 1990 and 2017, the unemployment rate of the labor force in Cypriot males was increased from 1.4 to 10.4% whereas in Cypriot females from 2.5 to 9.8% [33]. According to the 2003 and 2019 EU Health Surveys, the prevalence of smoking among Cypriot males decreased slightly from 38 to 32%, respectively. On the other hand, the prevalence of smoking for Cypriot females increased from 10% (in 2003) to 13% (in 2019) [34, 35]. In addition, the prevalence of heavy episodic drinking among the Cypriot population was decreased by 8% (2010–2016) [28]. This highlights the importance of both strengthening the social welfare policies as well as incorporating such policies in population mental health promotion strategies.

Age-standardized YLD and DALY rates due to eating disorders, such as anorexia nervosa and bulimia nervosa, have been identified as being higher in Cypriot females than males. The burden of eating disorders has traditionally been linked to body-image dissatisfaction and the role of social media [36, 37]. More research is needed in order to explore specific determinants for eating disorders in the Cypriot community. Nonetheless, the development and use of validated screening tests in primary healthcare setting may help to determine future health strategies regarding the burden of eating disorders in Cyprus.

Between 1990 and 2017, the leading cause of DALYs in elderly (70+ years) shifted to CVDs. A possible explanation for this is that most of the metabolic risk factors namely high fasting plasma glucose and/or high blood pressure are highly prevalent in the aged [38]. Over the 28-year study period, DALYs due to psoriasis in the elderly increased by 17%. The etiology of psoriasis involves interaction between genetic factors and exposure to smoking, alcohol drinking, and unhealthy dietary habits [39]. The association between CVDs and psoriasis and increased prevalence of cardiovascular risk factors have been described elsewhere [40]. The increasing impact of psoriasis among the elderly in Cyprus may be explained primarily by the shift towards unhealthy dietary habits and the high prevalence of NCDs, over the last decades. The interaction of multiple health conditions and risk factors prove a challenge for the prevention of NCDs in elderly. However, CVD risk assessment in elderly emerges an essential priority for health policy authorities.

### Strengths and limitations of the study

This study has several strengths and limitations. The present study has introduced the use of GBD 1990–2017 results to provide a comprehensive, up-to-date and in-depth overview of the burden of NCDs in terms of YLL, YLD, and DALY in Cyprus. A major strength of the GBD estimates is the internal consistency and comparability of the age-standardized YLL, YLD, and DALY estimates, which allow comparison across various countries and regions at multiple time points. Therefore, our findings are indispensable in helping Cypriot policy-makers to develop evidence-based prevention and intervention strategies for NCDs.

This study shares the limitations of the GBD 2017 study, which have been discussed in detail elsewhere [15, 41]. First and foremost, the GBD methodology produces sub-regional estimates for a number of countries; however, sub-regional estimates are not available for Cyprus. For Cyprus this means that the estimates presented here are based on the northern and the southern part of Cyprus combined. However, since the northern part is under Turkish occupation and the southern part is controlled by the government of Cyprus, there may be differences in health policy and prevention measures in the northern and southern part, which may impact NCD DALY rates and trends over time. Due to the unavailability of estimates for Cyprus, sub-regions variability across these sub-regions cannot be studied. Similarly, the GBD does not produce estimates for sub-groups of the population according to, for example, socio-economic status or ethnic background. Second, we did not analyze burden of NCDs by age groups, other than the 70+ age group, in detail, which would be necessary for the implementation of age-specific intervention strategies and/or activities in primary healthcare in Cyprus. Third, the NCD prevalence data in Cyprus is limited; the DALY estimates, in the GBD study, were informed by data from 32 data sources that consisted of health survey data and scientific literature reports. Both the low quality as well as possible low quality of these data sources may have introduced uncertainty and possible led to large UIs. The causes-of-death data in Cyprus, on the other hand, are predominantly provided by vital registration system. According to the GBD standard procedure, cause-of-death data are coded based on the International Classification of Disease (ICD) rules and the miscoded and non-specific coded deaths are re-assigned to specific cause-of-death categories. Therefore, there may be differences in the number of deaths by cause-of-death reported in the GBD 2017 study and those reported by the Cyprus statistical services.

### Implications for health policy in Cyprus

Our findings have important implications for evidence-based decision-making on the NCD intervention strategies in Cyprus. The majority of the NCDs share modifiable risk factors, namely tobacco use, hypertension, unhealthy diets, and alcohol abuse. Policymakers in Cyprus should consider targeting these NCD risk factors in targeted health prevention policies. Cyprus has a similar NCD risk profile compared with other Mediterranean countries (Greece, Italy, France, Spain, etc.) and the effect of preventive policies mainly for tobacco control has been evaluated [42, 43]. From this perspective, planning and developing patient-centered interventions of the NCD risk factors and/or early detection and disease screening can reduce the incidence of NCDs and exacerbation of prevalent NCDs. More importantly, the Cypriot health authorities should formulate and enact on prevention and health promotion strategies for NCDs in order to reduce population exposure in NCD risk factors. Furthermore, there is need to strengthen the epidemiological base for NCD prevalence in Cyprus.

## Conclusions

Despite a decrease in the burden of NCDs over the period from 1990 to 2017, NCDs are still a major public health challenge with CVDs, neoplasms, and musculoskeletal disorders to be major contributors to the burden of NCDs for both males and females. Implementation of early detection screening programmes of modifiable NCD risk factors and population-level health promotion programmes are needed to reduce the incidence and exacerbation of leading causes of NCDs in the Cypriot population.

## Supplementary Information

**Additional file 1.** Cause hierarchy for all non-communicable diseases.

**Additional file 2.** Age-standardized YLLs, YLDs, and DALYs and absolute changes for the main NCD drivers of change, Cypriot males and females, 1990–2017.

## Data Availability

The dataset used in this study is publically available at: http://ghdx.healthdata.org/gbd-results-tool.

## Abbreviations

CKD: Chronic Kidney Disease
COPD: Chronic Obstructive Pulmonary Disease
CVD: Cardiovascular Disease
DALY: Disability Adjusted Life Year
DW: Disability Weight
EU: European Union
GBD: Global Burden of Disease
ICD: International Classification of Disease
IHD: Ischemic Heart Disease
IHME: Institute for Health Metrics and Evaluation
NCD: Non-Communicable Disease
SMPH: Summary Measures of Population Health
SUDs: Substance Use Disorders
UI: Uncertainty Interval
WHO: World Health Organization
YLD: Years Lived with Disability
YLL: Years of Life Lost
