# Supplementary material for: Burden of non-communicable diseases in Cyprus, 1990–2017: findings from the Global Burden of Disease 2017 study
Source: Arch Public Health. 2021 Jul 29;79:138. doi: 10.1186/s13690-021-00655-8 (PMC8320095; doi:10.1186/s13690-021-00655-8)
Supplement: Supplementary file 1 — Additional file 1. Cause hierarchy for all non-communicable diseases. [file 13690_2021_655_MOESM1_ESM.docx]

**Additional file 1**: Cause hierarchy for all non-communicable diseases; GBD 2017 study*

| **Level 2** | **Level 3** | |  |
| --- | --- | --- | --- |
| **Neoplasms** | | | |
|  | Lip and oral cavity cancer | |  |
|  | Nasopharynx cancer | |  |
|  | Other pharynx cancer | |  |
|  | Esophageal cancer | |  |
|  | Stomach cancer | |  |
|  | Colon and rectum cancer | |  |
|  | Liver cancer | |  |
|  | Gallbladder and biliary tract cancer | |  |
|  | Pancreatic cancer | |  |
|  | Larynx cancer | |  |
|  | Tracheal, bronchus, and lung cancer | |  |
|  | Malignant skin melanoma | |  |
|  | Non-melanoma skin cancer | |  |
|  | Breast cancer | |  |
|  | Cervical cancer | |  |
|  | Uterine cancer | |  |
|  | Ovarian cancer | |  |
|  | Prostate cancer | |  |
|  | Testicular cancer | |  |
|  | Kidney cancer | |  |
|  | Bladder cancer | |  |
|  | Brain and nervous system cancer | |  |
|  | Thyroid cancer | |  |
|  | Mesothelioma | |  |
|  | Hodgkin lymphoma | |  |
|  | Non-Hodgkin lymphoma | |  |
|  | Multiple myeloma | |  |
|  | Leukemia | |  |
|  | Other malignant neoplasms | |  |
|  | Other neoplasms | |  |
| **Cardiovascular diseases** | | |  |
|  | Rheumatic heart disease | |  |
|  | Ischemic heart disease | |  |
|  | Stroke | |  |
|  | Hypertensive heart disease | |  |
|  | Non-rheumatic valvular heart disease | |  |
|  | Cardiomyopathy and myocarditis | |  |
|  | Atrial fibrillation and flutter | |  |
|  | Aortic aneurysm | |  |
|  | Peripheral artery disease | |  |
|  | Endocarditis | |  |
|  | Other cardiovascular and circulatory diseases | |  |
| *(continued from previous page)* | |  |  |
| **Level 2** | | **Level 3** |  |
| **Chronic respiratory diseases** | | |  |
|  | Chronic obstructive pulmonary disease | |  |
|  | Pneumoconiosis | |  |
|  | Asthma | |  |
|  | Interstitial lung disease and pulmonary sarcoidosis | |  |
|  | Other chronic respiratory diseases | |  |
| **Digestive diseases** | | |  |
|  | Cirrhosis and other chronic liver diseases | |  |
|  | Upper digestive system diseases | |  |
|  | Appendicitis | |  |
|  | Paralytic ileus and intestinal obstruction | |  |
|  | Inguinal, femoral, and abdominal hernia | |  |
|  | Inflammatory bowel disease | |  |
|  | Vascular intestinal disorders | |  |
|  | Gallbladder and biliary diseases | |  |
|  | Pancreatitis | |  |
|  | Other digestive diseases | |  |
| **Neurological disorders** | | |  |
|  | Alzheimer's disease and other dementias | |  |
|  | Parkinson's disease | |  |
|  | Epilepsy | |  |
|  | Multiple sclerosis | |  |
|  | Motor neuron disease | |  |
|  | Headache disorders | |  |
|  | Other neurological disorders | |  |
| **Mental disorders** | | |  |
|  | Schizophrenia | |  |
|  | Depressive disorders | |  |
|  | Bipolar disorder | |  |
|  | Anxiety disorders | |  |
|  | Eating disorders | |  |
|  | Autism spectrum disorders | |  |
|  | Attention-deficit/hyperactivity disorder | |  |
|  | Conduct disorder | |  |
|  | Idiopathic developmental intellectual disability | |  |
|  | Other mental disorders | |  |
| **Substance use disorders** | | |  |
|  | Alcohol use disorders | |  |
|  | Drug use disorders | |  |
| **Diabetes and kidney diseases** | | |  |
|  | Diabetes mellitus | |  |
|  | Chronic kidney disease | |  |
|  | Acute glomerulonephritis | |  |
| *(continued from previous page)* | | |  |
| **Level 2** | **Level 3** | |  |
| **Skin and subcutaneous diseases** | | |  |
|  | Dermatitis | |  |
|  | Psoriasis | |  |
|  | Bacterial skin diseases | |  |
|  | Scabies | |  |
|  | Fungal skin diseases | |  |
|  | Viral skin diseases | |  |
|  | Acne vulgaris | |  |
|  | Alopecia areata | |  |
|  | Pruritus | |  |
|  | Urticaria | |  |
|  | Decubitus ulcer | |  |
|  | Other skin and subcutaneous diseases | |  |
| **Sense organ diseases** | | |  |
|  | Blindness and vision impairment | |  |
|  | Age-related and other hearing loss | |  |
|  | Other sense organ diseases | |  |
| **Musculoskeletal disorders** | | |  |
|  | Rheumatoid arthritis | |  |
|  | Osteoarthritis | |  |
|  | Low back pain | |  |
|  | Neck pain | |  |
|  | Gout | |  |
|  | Other musculoskeletal disorders | |  |
| **Other non-communicable diseases** | | |  |
|  | Congenital birth defects | |  |
|  | Urinary diseases and male infertility | |  |
|  | Gynecological diseases | |  |
|  | Hemoglobinopathies and hemolytic anemias | |  |
|  | Endocrine, metabolic, blood, and immune disorders | |  |
|  | Oral disorders | |  |
|  | Sudden infant death syndrome | |  |
| * Institute for Health Metrics and Evaluation. GBD 2017 causes of disease and injuries. Seattle: University of Washington; 2018 <http://www.healthdata.org/sites/default/files/files/Projects/GBD/GBD_2017_heirarchies.zip> | | |  |
