# Supplementary material for: Burden of non-communicable diseases in Cyprus, 1990–2017: findings from the Global Burden of Disease 2017 study
Source: Arch Public Health. 2021 Jul 29;79:138. doi: 10.1186/s13690-021-00655-8 (PMC8320095; doi:10.1186/s13690-021-00655-8)
Supplement: Supplementary file 2 — Additional file 2. Age-standardized YLLs, YLDs, and DALYs and absolute changes for the main NCD drivers of change, Cypriot males and females, 1990–2017. [file 13690_2021_655_MOESM2_ESM.docx]

**Additional file 2**:

Age-standardized YLLs, YLDs, and DALYs and absolute changes for the main NCD drivers of change, Cypriot males and females, 1990 – 2017

| **Largest decreases by NCD cause, Cypriot males, 1990 – 2017** | | | | | | | | | | | | | | | | | | | | | | | |  |
| --- | --- | --- | --- | --- | --- | --- | --- | --- | --- | --- | --- | --- | --- | --- | --- | --- | --- | --- | --- | --- | --- | --- | --- | --- |
|  | **YLLs** | | | | | | |  | **YLDs** | | | | | |  | | **DALYs** | | | | | | | |
|  | 1990 | | | 2017 | | Absolute age-standardized  YLL changes | |  | 1990 | | 2017 | | Absolute age-standardized  YLD changes | |  | | 1990 | | 2017 | | | Absolute age-standardized  DALY changes | | |
| IHD | 4,030  (3,806 – 4,249) | | | 2,117  (1,872 – 2,398) | | - 1,913 | | Oral disorders | 244  (150 – 371) | | 209  (126 – 322) | | - 35 | | IHD | | 4,141  (3,908 –  4,367) | | 2,197  (1,943 – 2,476) | | | - 1,944 | | |
| Stroke | 1,113  (1,002 – 1,231) | | | 507  (447 – 582) | | - 606 | | IHD | 110  (77 – 151) | | 79  (55 – 108) | | - 31 | | Stroke | | 1,250  (1,137 – 1,375) | | 635  (556 – 712) | | | - 615 | | |
| Congenital birth defects | 708  (448 – 991) | | | 130  (106 – 156) | | - 578 | | Asthma | 196  (129 – 280) | | 173  (113 – 247) | | - 23 | | Congenital birth defects | | 882  (608 – 1,178) | | 302  (247 – 366) | | | - 580 | | |
| Diabetes | 604  (534 – 689) | | | 387  (335 – 445) | | - 217 | | Blindness and vision impairment | 186  (120 – 277) | | 164  (106 – 244) | | - 22 | | COPD | | 807  (714 – 913) | | 615  (540 – 703) | | | - 192 | | |
| COPD | 559  (481 – 650) | | | 374  (320 – 456) | | - 185 | | Depressive disorders | 331  (233 – 451) | | 321  (277 – 439) | | - 10 | | Diabetes | | 1,059  (887 – 1,258) | | 935  (754 – 1,155) | | | - 124 | | |
| **Largest increases by NCD cause, Cypriot males, 1990 – 2017** | | | | | | | | | | | | | | | | | | | | | | | |  |
| Pancreatic cancer | 124  (103 – 164) | | | 179  (151 – 210) | | + 55 | | Diabetes | 455  (313 – 637) | | 548  (374 – 763) | | + 93 | | Pancreatic cancer | | 125  (104 – 166) | | 180  (153 – 212) | | | + 55 | | |
| Drug use disorders | 51  (37 – 63) | | | 91  (74 – 111) | | + 40 | | Prostate cancer | 22  (15 – 20) | | 56  (38 – 83) | | + 34 | | Drug use disorders | | 174  (136 – 214) | | 227  (182 – 272) | | | + 53 | | |
| Kidney cancer | 31  (26 – 49) | | | 56  (46 – 67) | | + 25 | | Drug use  disorders | 124  (86 – 165) | | 136  (95 – 180) | | + 12 | | Acne vulgaris | | 45  (27 – 72) | | 76  (46 – 122) | | | + 31 | | |
| Liver cancer | 100  (86 – 124) | | | 119  (98 – 141) | | + 19 | | Colorectal cancer | 10  (7 – 14) | | 21  (14 - 28) | | + 11 | | Kidney cancer | | 32  (27 – 50) | | 58  (46 – 70) | | | + 26 | | |
| Urinary diseases | 41  (35 – 49) | | | 55  (47 – 64) | | + 14 | | Alcohol use disorders | 137  (91 – 197) | | 145  (98 – 207) | | + 8 | | Psoriasis | | 127  (90 – 169) | | 152  (108 – 202) | | | + 25 | | |
| *(continued from previous page)* | | | | | | | | | | | | | | | | | | | | | | |  |  |
| **Largest decreases by NCD cause, Cypriot females, 1990 – 2017** | | | | | | | | | | | | | | | | | | | | | | |  |  |
|  | | **YLLs** | | | | |  | | | **YLDs** | | | | | |  | | **DALYs** | | | | |  |  |
|  | | 1990 | 2017 | | Absolute age-standardized  YLL changes | |  | | | 1990 | | 2017 | | Absolute age-standardized  YLD changes | |  | | 1990 | | 2017 | Absolute age-standardized  DALY changes | |  |  |
| IHD | | 1,727  (1,610 – 1,856) | 602  (533 – 709) | | - 1,125 | | Oral disorders | | | 302  (190 – 451) | | 259  (161 – 391) | | - 43 | | IHD | | 1,798  (1,675 – 1,932) | | 648  (579 – 752) | - 1,150 | |  |  |
| Stroke | | 969  (877 – 1,065) | 323  (283 – 381) | | - 646 | | Hemoglobinopathies | | | 67  (38 – 107) | | 34  (18 – 58) | | - 33 | | Stroke | | 1,108  (1,007 – 1,216) | | 451  (396 – 512) | - 657 | |  |  |
| Congenital birth defects | | 595  (415 – 833) | 123  (101 – 148) | | - 472 | | Gynecological diseases | | | 250  (171 – 355) | | 222  (149 – 314) | | - 28 | | Diabetes | | 939  (779 – 1,120) | | 597  (460 – 755) | - 342 | |  |  |
| Diabetes | | 558  (473 – 653) | 199  (170 – 231) | | - 361 | | COPD | | | 237  (195 – 284) | | 212  (175 – 253) | | - 25 | | COPD | | 484  (402 – 554) | | 343  (293 – 394) | - 141 | |  |  |
| Breast cancer | | 602  (498 – 709) | 446  (371 – 526) | | - 156 | | IHD | | | 71  (48 – 96) | | 46  (31 – 64) | | - 25 | | Breast cancer | | 644  (534 – 755) | | 511  (427 – 604) | - 133 | |  |  |
| **Largest increases by NCD cause, Cypriot females, 1990 – 2017** | | | | | | | | | | | | | | | | | | | | | | |  |  |
| Lung cancer | | 158  (134 – 190) | 177  (152 – 203) | | + 19 | | Acne vulgaris | | | 61  (36 – 98) | | 103  (62 – 164) | | + 42 | | Acne vulgaris | | 61  (36 – 98) | | 103  (62 – 164) | + 42 | |  |  |
| Pancreatic cancer | | 73  (65 – 82) | 91  (78 – 106) | | + 18 | | Psoriasis | | | 134  (94 – 176) | | 161  (115 – 213) | | + 27 | | Psoriasis | | 134  (94 – 176) | | 162  (115 – 213) | + 28 | |  |  |
| Motor neuron disease | | 5  (2 – 8) | 9  (2 – 12) | | + 4 | | Eating disorders | | | 126  (82 – 188) | | 151  (98 – 255) | | + 25 | | Eating disorders | | 126  (82 – 188) | | 151  (98 – 225) | + 25 | |  |  |
| Kidney cancer | | 15  (13 – 19) | 18  (15 – 22) | | + 3 | | Breast cancer | | | 41  (27 – 57) | | 64  (43 – 90) | | + 23 | | Lung cancer | | 160  (135 – 192) | | 179  (154 – 206) | + 19 | |  |  |
| Alcohol use disorders | | 3  (2 – 7) | 4  (3 – 6) | | + 1 | | Diabetes | | | 380  (256 – 533) | | 399  (266 – 562) | | + 19 | | Pancreatic cancer | | 74  (65 – 83) | | 92  (79 – 107) | + 18 | |  |  |
| COPD: Chronic obstructive pulmonary disease; DALYs: Disability-adjusted life years; IHD: Ischemic heart disease; NCD: Non-communicable disease; YLs: Years of life lost; YLDs: Years lived with disability | | | | | | | | | | | | | | | | | | | | | | |  |  |
